# Epigenetic silencing of serine protease HTRA1 drives polyploidy

**DOI:** 10.1186/s12885-016-2425-8

**Published:** 2016-07-07

**Authors:** Nina Schmidt, Inga Irle, Kamilla Ripkens, Vanda Lux, Jasmin Nelles, Christian Johannes, Lee Parry, Kirsty Greenow, Sarah Amir, Mara Campioni, Alfonso Baldi, Chio Oka, Masashi Kawaichi, Alan R. Clarke, Michael Ehrmann

**Affiliations:** Centre for Medical Biotechnology, Faculty of Biology and Geography, University Duisburg-Essen, Universitaetsstrasse, D-45117 Essen, Germany; School of Biosciences, Cardiff University, Cardiff, CF10 3US UK; Department of Biochemistry and Biophysics, Section of Pathology, Second University of Naples, 80100 Naples, Italy; Division of Gene Function in Animals, Nara Institute of Science and Technology, 8916-5 Takayama, Ikoma, Nara 630-0192 Japan

**Keywords:** HTRA1, MDB2, serine protease

## Abstract

**Background:**

Increased numbers and improperly positioned centrosomes, aneuploidy or polyploidy, and chromosomal instability are frequently observed characteristics of cancer cells. While some aspects of these events and the checkpoint mechanisms are well studied, not all players have yet been identified. As the role of proteases other than the proteasome in tumorigenesis is an insufficiently addressed question, we investigated the epigenetic control of the widely conserved protease HTRA1 and the phenotypes of deregulation.

**Methods:**

Mouse embryonal fibroblasts and HCT116 and SW480 cells were used to study the mechanism of epigenetic silencing of *HTRA1*. In addition, using cell biological and genetic methods, the phenotypes of downregulation of HTRA1 expression were investigated.

**Results:**

*HTRA1* is epigenetically silenced in HCT116 colon carcinoma cells via the epigenetic adaptor protein MBD2. On the cellular level, HTRA1 depletion causes multiple phenotypes including acceleration of cell growth, centrosome amplification and polyploidy in SW480 colon adenocarcinoma cells as well as in primary mouse embryonic fibroblasts (MEFs).

**Conclusions:**

Downregulation of HTRA1 causes a number of phenotypes that are hallmarks of cancer cells suggesting that the methylation state of the *HtrA1* promoter may be used as a biomarker for tumour cells or cells at risk of transformation.

**Electronic supplementary material:**

The online version of this article (doi:10.1186/s12885-016-2425-8) contains supplementary material, which is available to authorized users.

## Background

Mammalian HtrA1 belongs to the widely conserved high-temperature requirement A (HtrA) family of homo-oligomeric serine proteases that are implicated in protein quality control. The ubiquitously expressed HTRA1 is composed of a signal sequence for secretion, a partial insulin like growth factor binding protein-7 domain of unknown function, a serine protease domain resembling chymotrypsin and one C-terminal PDZ domain. HTRA1 has been shown to have at least three cellular locations. The extracytoplasmic pool is involved in the homeostasis of the extracellular matrix as HTRA1 degrades fibronectin, fibromodulin, aggrecan and decorin. In addition, intracellular HTRA1 localizes to microtubules or to the nucleus (for review see [1]).

Human HTRA1 has been implicated in several severe pathologies including cancer, age-related macular degeneration, Alzheimer’s disease, arthritis and familial ischemic cerebral small-vessel disease [1]. In many of these diseases, protein fragments or aggregates are either causative for disease or are disease modifying factors that are produced or degraded by HTRA1. Furthermore, several publications link HTRA1 to tumorigenesis as its gene has been found to be downregulated in many tumours [2], and forcing its re-expression interfered with proliferation of metastatic melanoma cells [3] and cell migration [4], suggesting a tumour suppressor function. In addition, HTRA1 was shown to modulate cisplatin- and paclitaxel-induced cytotoxicity and low levels of HTRA1 correlated with a poor response to drug treatment whilst higher levels of HTRA1 correlated with a higher response rate [5]. Downregulation of the *HTRA1* gene in tumour cells has been linked with epigenetic mechanisms [2, 6] and the *HTRA1* promoter was identified as a target of the histone deacetylase HDAC1 [7]. Despite these recent advances, the function and mechanism of silencing of intracellular HTRA1 underlying its involvement in cell proliferation, migration and tumorigenesis are currently not well understood.

We show that *HTRA1* is epigenetically silenced in HCT116 colon carcinoma cells and during early stages of tumorigenesis in a mouse model of intestinal cancer. Downregulation of HTRA1 causes a multiple phenotypes that are hallmarks of cancer cells including increased proliferation of mouse embryonic fibroblasts (MEF), as well as chromosome and centrosome amplifications.

## Methods

### Cell lines and drug treatments

This study received ethical approval from Cardiff University’s Animal Welfare and Ethical Review Body (previously known as the ERP), and all animal procedures were conducted in accordance with UK Home Office regulations. HCT116, SW480 cells and MEFs were maintained in Dulbecco’s modified Eagle’s medium (DMEM) supplemented with 10 % fetal bovine serum, 1 % penicillin and 1 % streptomycin at 37 °C in humidified atmosphere with 5 % CO_2_. MEFs were isolated from E13.5 and E14.5 embryos derived from four different breedings. *Htra1*^*−/−*^ mice were described previously [8]. SW480 and HCT116 cells were obtained from ATCC.

Cells were seeded at a low density for 16 h and were treated with indicated concentrations of 5-Aza-dC (Sigma) or 400 nM TSA (NEB) for 16 h. For drug combination cells were treated with 5-Aza-dC for 48 h followed by TSA for additional 16 h.

### Oligonucleotides

All oligonucleotides used are listed in Additional file 1: Table S1.

### Lentiviral preparation and viral infection

Hairpin sequences directed against *HTRA1* or *MBD2* were cloned into the lentiviral pLKO.1puro vector using *AgeI* and *EcoRI*. 293 T cells were transfected with lentiviral vectors encoding shRNAs (sh*HTRA1* D3 and S8) or nonsense RNA (EV ctrl.) and lentiviral packaging vectors pCMVΔR8.2 (*gag pol*) and pHITG (*env*). Viruses were collected 48 h after transfection. HCT116 and SW480 cells were infected with the collected viruses twice over 18 h in the presence of polybrene. Infected cells were selected using 1.6 μg/ml puromycin.

### Confocal laser microscopy and antibodies

24 h after plating, cells were stained against α-tubulin (Invitrogen), β-tubulin (Molecular Probes), γ-tubulin (Sigma-Aldrich) or Actin (MP Biomedicals). For detection, secondary antibodies conjugated with Alexa-488 or Phalloidin-TRITC (Molecular Probes) were used. Nuclei were stained with DAPI (Molecular Probes). Samples were analysed in a Leica TCS SL (SP5) laser confocal microscope and Leica Confocal Software was used for imaging. Images were taken using an HCX PL APO x 63 oil objective lens.

### RNA purification and quantitative real-time-PCR (qRT-PCR) analysis

RNA purification and qRT-PCR were done as described [9]. All mRNA levels were normalized to mRNA levels of the “house-keeping” gene *GAPDH* for samples from human cell lines or *β-actin* for samples from murine cell lines to obtain the mean normalized expression. Analysis of data sets was carried out with Q-Gene software [10].

### Karyotyping of MEFs and SW480 cells

Exponentially growing SW480 (Parental, EV ctrl. and sh*HTRA1* D3 and S8) and MEF cultures were incubated in N-deacetyl-N-methylcolchicine (Colcemid; 0.08 μg/ml) for 2 h to arrest mitotic cells in highly condensed metaphase like stages. Monolayers were rinsed and centrifuged for 5 min at 120 g. Cell sediments were hypotonically treated with 5 ml of 75 mM KCl for 10 min. Following centrifugation the swollen cells were gently mixed with 5 ml of fixing solution (methanol/acetic acid; 3/1), centrifuged, and again mixed with fixing solution. Cell suspensions were dropped onto pre-cleaned, wet, ice-cold glass microscope slides to obtain good spreading of the chromosome sets. After air-drying overnight, the preparations were stained in Giemsa-solution (5 %). Intact metaphase cells were counted for their chromosome numbers at 1000 fold magnification (oil-immersion).

### Bisulfite modification and bisulfite sequencing PCR (BSP) of genomic DNA

Genomic DNA was prepared from cell lines or murine colon polyp cells using QIAamp DNA Mini Kit (Qiagen). Bisulfite conversion of 2 μg genomic DNA was performed using the EpiTect Bisulfite Kit (Qiagen). Origin of polyps: no. 13 from mouse no. 444, no. 18 from mouse no. 508, no. 22 from mouse no. 509, nos. 97, 98, 99, 101 from mouse no. 1122 and nos. 145 & 147 from mouse no. 495. 3 μl of bisulfite treated genomic DNA were used for PCR amplification. PCR products were purified and cloned into pCR2.1-TOPO using TOPO TA Cloning Kit (Invitrogen). DNA was sequenced and methylation status of the DNA sequences was analysed using BIQAnalyzer [11].

### Chromatin immunoprecipitation (ChIP)

Confluent SW480 and HCT116 cells were used for ChIP experiments. For immunoprecipitation, 2 μg of RNApolII (Active Motif, No. 39097), IgG (Active Motif), H3 (Abcam, No. 1791), H3K9ac (Diagenode, pAB-177-050) and 10 μg MBD2a/b (Sigma, M7318) antibodies were used. qRT-PCR was used to determine the enrichment of immunoprecipitated DNA relative to the input material using gene-specific (*HTRA1*) and control (*GAPDH*) primer sets (Additional file 1: Table S1). For more details see Additional file 1.

### Protein purification

HTRA1 was purified as described [12]. Purified HTRA1 was dialyzed against 50 mM Tris HCl, pH 8.0, 150 mM NaCl and stored at −70 °C. 6His tagged MBD2b, pET28MBD2b were purified using Protino Ni-TED 2000 column (Macherey-Nagel) following manufacturer’s instruction. Subsequently, MBD2b fractions were dialyzed against 50 mM NaH_2_PO_4_, pH 8.0 and stored at −70 °C.

### EMSA

Electrophoretic Mobility Shift Assay was done in 10 μl of EMSA-buffer (50 mM Tris, 5 mM MgCl_2_, 10 mM DTT, pH 7.5) for 5 min at RT. Reaction mixtures were loaded on a TBE-gel which was stained with ethidium bromide.

### Protease protection assay

Trypsin (Sigma) digests of MBD2b were performed by incubating 5 μg MBD2b with various amounts of trypsin for 20 min at 37 °C in reaction buffer (50 mM Tris, 5 mM MgCl_2_, pH 7.5). To analyse the effect of DNA-binding, MBD2b was pre-incubated with DNA-oligonucleotides (Additional file 1: Table S1) at equimolar concentration for 10 min at RT before adding trypsin.

### Statistical analyses

Statistical analyses were carried out using GraphPad Prism5 software (GraphPad Software). Gaussian distribution of data sets was tested via D’Agostino and Pearson omnibus normality test or (for smaller n) via Komologrov Smirnov normality test with alpha = 0.05. Data sets following a Gaussian distribution were analysed by a two-tailed t-test if variance homogeneity was given. A two-tailed Mann–Whitney U test was used for analysing data sets not following a Gaussian distribution or with significant difference in variances (Levene-test *p*-value <0.2). For analysis of nominal data sets Fisher’s exact test was performed.

## Results

### HTRA1 is epigenetically silenced in HCT116 cells and in polyps arising in Apc^Min+^ mice

**Fig. 1:**
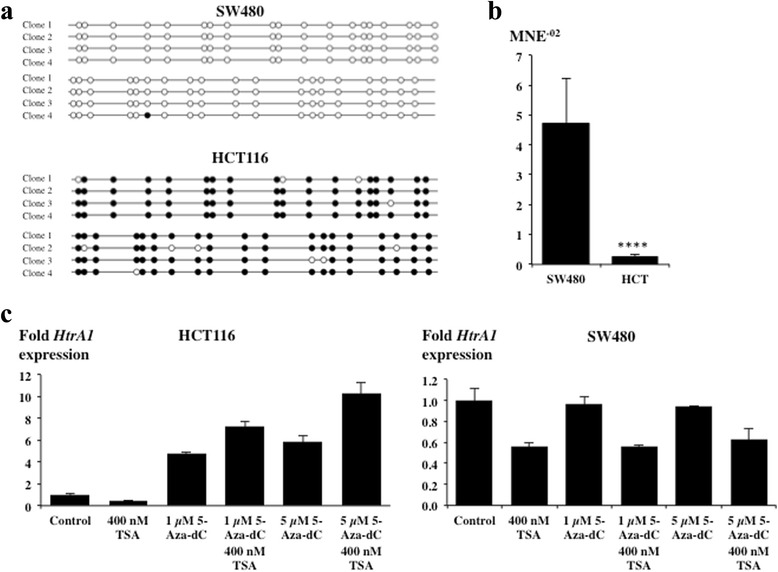
Epigenetic regulation of *HTRA1* expression in HCT116 cells. **a** Methylation of the human *HTRA1* promoter (−388 bp to −115 bp) was determined by sequencing 4 independent clones of bisulfite treated DNA of HCT116 and SW480 cells. **b** Mean normalized expression (MNE) of *HTRA1* in SW480 and HCT116 cells determined by qRT-PCR (n = 3 independent cell lines, each done in triplicates, two-tailed Mann–Whitney U test, p-value <0.0001). **c** *HTRA1* mRNA levels in HCT116 and SW480 cells following treatment with 5-Aza-dC and TSA determined by qRT-PCR. Results of one representative experiment are shown. MNE of control was set to 1; standard deviation was calculated from triplicates

As *HTRA1* is downregulated in a range of tumours we determined if silencing of *HTRA1* occurs in cancer cell lines by analysing methylation of the *HTRA1* promoter in two colon carcinoma cell lines (HCT116 and SW480). These data indicate that the *HTRA1* promoter is methylated in HCT116 cells but not in SW480 cells and that promoter methylation status correlates with reduced the expression levels of the *HTRA1* gene (Fig. 1a, b). To obtain further evidence for epigenetic silencing of the *HTRA1* promoter, HCT116 and SW480 cells were treated individually or in combination with the DNA methyltransferase (DNMT) inhibitor 5-aza-deoxycytidine (5-aza-dC) and Trichostatin A (TSA), an inhibitor of histone deacetylases (HDAC). Incorporation of 5-aza-dC into DNA inhibits methylation at CpG islands by DNA methyltransferase whereas Trichostatin A inhibits histone deacetylation by HDAC [13, 14]. Therefore, both inhibitors promote gene expression. Consequently, changes in *HTRA1* expression were detected following treatment of HCT116 cells with either 1 μM 5-Aza-dC in combination with 400 nM TSA or 5 μM 5-Aza-dC in combination with 400 nM TSA, leading to 7 and 10 fold higher levels of expression of *HTRA1*, respectively (Fig. 1c). In contrast, no increases were observed in SW480 cells (Fig. 1c). In this cell line, exposure to TSA even reduced *HTRA1* expression, which may be due to the side effect of TSA causing cell cycle arrest in colon carcinoma cells and fibroblasts [15, 16].

**Fig. 2:**
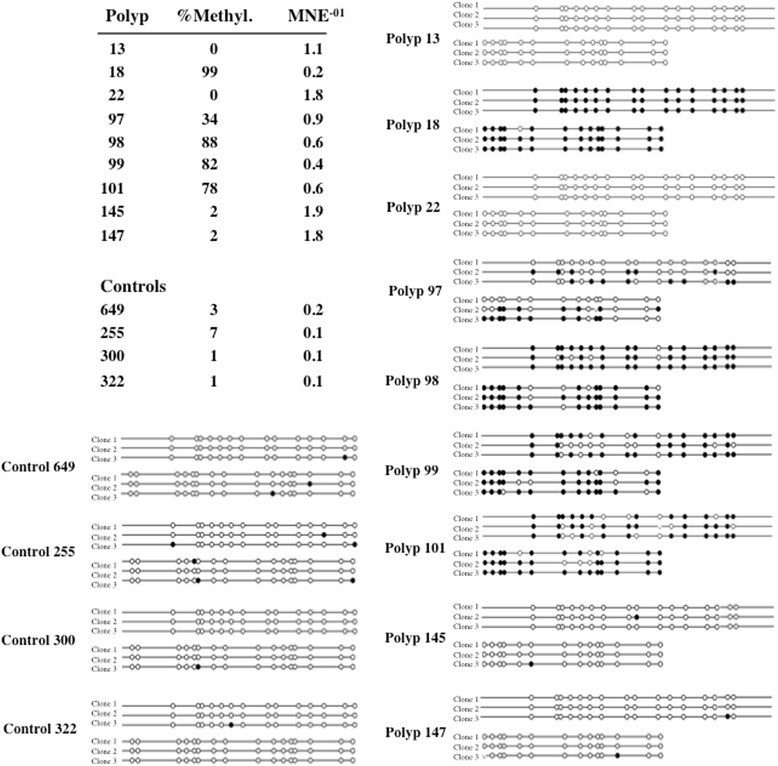
*Htra1* expression and methylation of the *Htra1* promoter in polyps. Mean normalized expression (MNE) of *Htra1* in polyps isolated from *Apc* ^*Min+*^ mice was determined by qRT-PCR. Methylation of the murine *Htra1* promoter (−252 bp to −12 bp) was determined by sequencing 3 independent clones of bisulfite treated DNA isolated from the same polyps that were used for measuring mRNA levels by qRT-PCR. Controls are four independent samples taken from large intestine

To obtain *in vivo* evidence, we analysed *Htra1* expression in the intestines of *Apc*^*Min+*^ mice, a well established model of early events in intestinal tumorigenesis [17], and in polyps of colon adenomas arising in these animals. In the normal intestine samples, *Htra1* expression was very low but epigenetic silencing was not involved as none of the samples contained a methylated CpG island (Fig. 2, controls). In contrast, *Htra1* expression was differentially regulated during polyp formation and the level of *Htra1* expression correlated with the degree of DNA methylation (Fig. 2). Of 9 colonic polyps analysed, the *Htra1* promoter was unmethylated in 4 polyps (polyps 13, 22, 145 and 147), methylated in 4 polyps (polyps 18, 98, 99 and 101) and partially methylated in one polyp (polyp 97). These data suggest that silencing of *Htra1* is occurring during tumorigenesis *in vivo*.

#### Methyl Binding Domain protein 2 (MBD2) mediates epigenetic silencing of Htra1

**Fig. 3:**
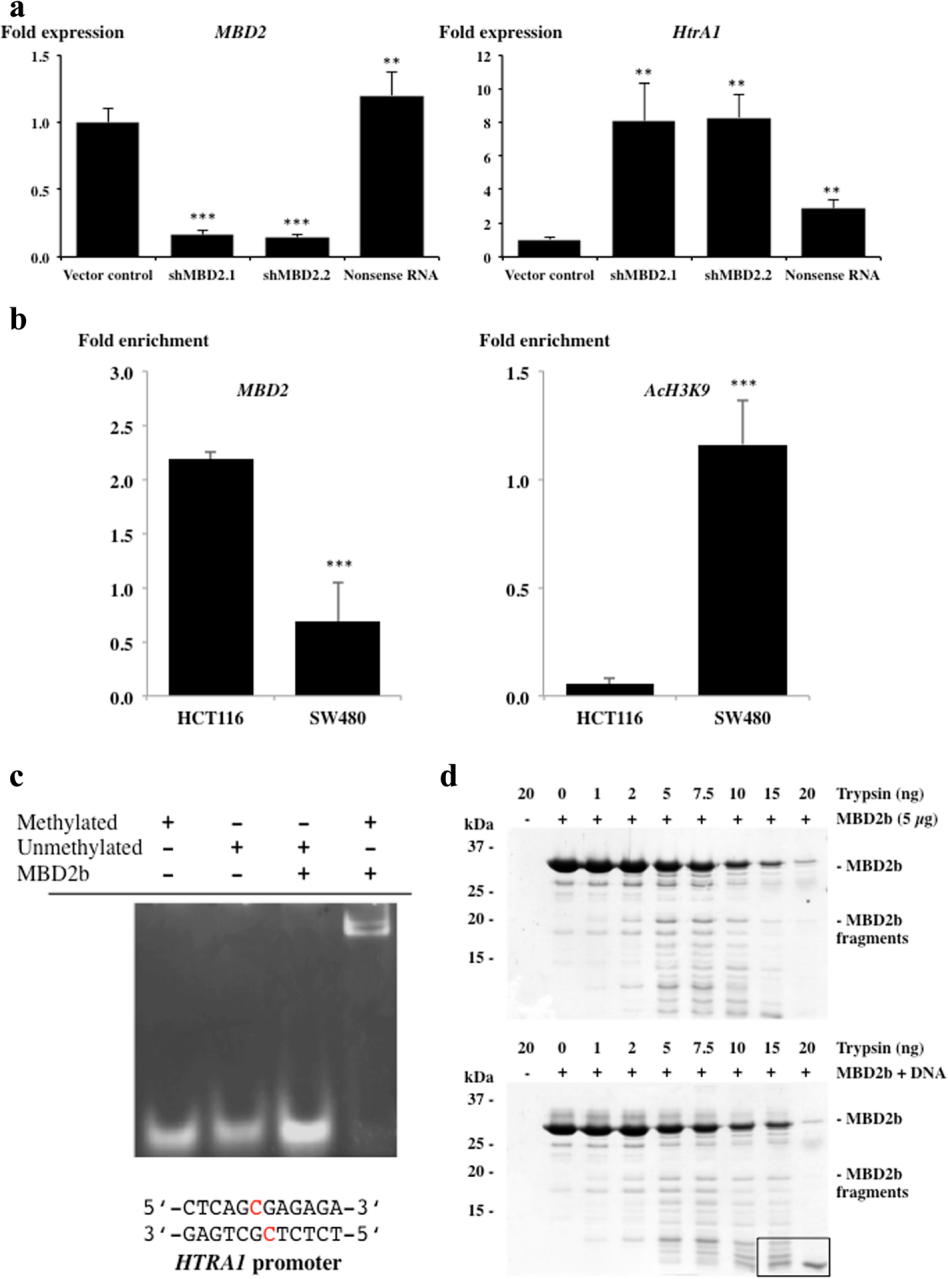
MDB2 mediates silencing of the *HTRA1* promoter. **a** Fold expression of *MBD2* and *HTRA1* in HCT116 cells stably downregulated by different shRNAs (1 and 2) or random RNA sequence (nonsense RNA) compared to the vector control (set to 1). Standard deviation is indicated (n ≥ 2 independent experiments done in triplicates, two-tailed Mann–Whitney U test, *p*-values *HTRA1* expression: sh*MBD2.1* = 0.0039, sh*MBD2.2* = 0.0028, Nonsense RNA = 0.0028; *p*-values *MBD2* expression: sh*MBD2.1* = 0.0002, sh*MBD2.2* = 0.0002, Nonsense RNA = 0.0034)). **b** ChIP analyses of MBD2 binding the human *HTRA1* promoter (−453 bp to −336 bp) in HCT116 and SW480 cells. Results are expressed as fold enrichment compared to the reference *GAPDH* promoter (*n* = 4, 2 independent chromatin fractions, unpaired two-tailed t-test, *p*-value <0.001) (left). ChIP analyses of H3K9 acetylation at the human *HTRA1* promoter (−453 bp to −336 bp). Results are expressed as fold enrichment compared to the reference histone 3 (*n* = 3, 2 independent chromatin fractions, unpaired two-tailed t-test, *p*-value <0.001) (right). **c** Electric mobility shift assay using methylated or unmethylated 12 bp double stranded oligonucleotide from the CpG island of the *HTRA1* promoter and purified MBD2b in equimolar concentrations (70 pmol). MBD2-DNA complex formation was analysed on 12 % agarose gels. **d** Proteolysis of MBD2b by trypsin. 5 μg of purified MBD2b were incubated with the amounts of trypsin indicated for 20 min at 37 °C. Samples were analysed on SDS PAGE (upper panel). Proteolysis of MBD2b with bound methylated oligonucleotide by trypsin. The protected MBD2b fragment is boxed (lower panel)

Having established epigenetic silencing of *HTRA1 in vivo* and *in vitro*, we wished to obtain a deeper insight by identifying factors that are involved in this process. Since MBD2 has been shown to alter tumorigenesis in *Apc*^*Min+*^ mice [18], we tested whether MBD2 mediates silencing of *HTRA1* by stable knockdown of MBD2. HCT116 cells were used for these experiments because the *HTRA1* promoter is epigenetically silenced in these cells. Knockdown was done using two independent shRNAs, resulting in an about 5 fold reduction of MBD2 expression which led to a 5 (sh*MBD2.1*) and 10 fold (sh*MBD2.2*) increase in *HTRA1* gene expression (Fig. 3a). This effect was specific to MBD2 as levels of *MBD1* and *MeCP1* were unaffected by the knockdown of MBD2 expression (not shown). These findings expand published data that identified HTRA1 as a target of mouse HDAC1 [7].

To analyse methylation-dependent binding of MBD2 to the *HTRA1* promoter we performed ChIP assays in HCT116 and SW480 cells. Primer sets were designed covering the *HTRA1* promoter sequence of the methylation analysis shown in Fig. 1a. The specificity of ChIP assays was verified by using nonspecific IgG (binding < 0.02 %). As a positive control, the *GAPDH* promoter was shown to be equally associated with RNAPolII (data not shown). The occupancy of MBD2 was up to 3-fold higher in HCT116 compared to SW480 cells (Fig. 3b), whereas the acetylation of Lys9 at histone 3, a chromatin marker for transcriptional activity, displayed a reversed pattern (Fig. 3b). Similar results were published in HCT116 cells, where binding of MBD2 to the *TFF2* promoter was detected along with reduced histone acetylation levels [19]. To obtain further evidence for MBD2 binding to the *HTRA1* promoter we purified recombinant MBD2b, a truncated version of MBD2 lacking the N-terminal 149 residues. This variant mediates silencing of a target promoter in cell culture [20]. To obtain a first indication of the affinity of MBD2 to the *HTRA1* promoter we performed electrophoretic mobility shift assays using a specific sequence of 12 nucleotides occurring at position −376 bp to −364 bp in the CpG island of the *HTRA1* promoter. These data indicated that the 12mer nucleotide bound in 1:1 stoichiometry to MBD2 in its methylated but not in its unmethylated form (Fig. 3c). These results are in agreement with published data reporting that MBD2 binds to synthetic 34mer nucleotides with high affinity [21]. To obtain independent evidence for binding of MBD2 to *HTRA1* promoter DNA, we performed proteolytic digests of MBD2 without or with bound DNA (Fig. 3d). This strategy was chosen as ligand binding commonly causes conformational changes that lead to increased stability of the protein in protease assays. Trypsin digests MBD2 in the presence and absence of DNA. However, in the presence of DNA, several fragments of about 6–10 kDa are protected (Fig. 3d). The size of the prominent band corresponds to the size of the DNA binding domain of MBD2 (residues 24 through 86). Together, these data therefore confirm that MBD2 binds to, and epigenetically regulates, *HTRA1* in HCT116 cells.

### Loss of HTRA1 causes accelerated proliferation

**Fig. 4:**
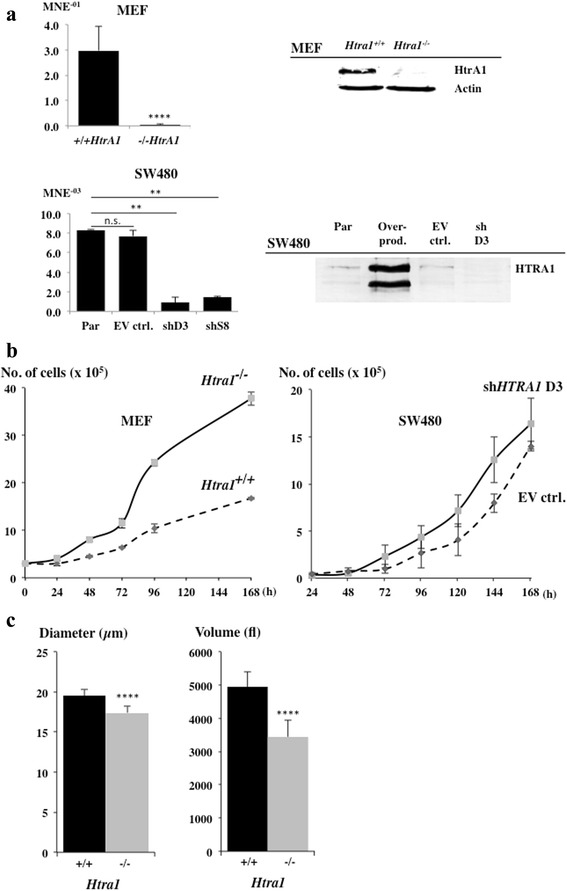
Increased proliferation after *HTRA1* depletion. **a** HTRA1 expression measured by qRT-PCR (*n* = 2 independent experiments, each assayed in triplicate) and Western blots. Upper panel, *Htra1* ^*+/+*^ and *Htra1* ^*−/−*^ MEFs (two-tailed Mann–Whitney U test, *p*-value <0.0001). Lower panel, supernatants of parental or stably transfected SW480 cells (Par, untransfected SW480; overprod., SW480 stably transfected with plasmid p50HTRA1; EV ctrl., corresponding empty vector control; shD3, downregulated via sh*HTRA1* D3; shS8, downregulated via sh*HTRA1* S8; two-tailed Mann–Whitney U test, *p*-values: EV ctrl. = 0.3776, shD3 = 0.005, shS8 = 0.005). Actin was used as loading control for cell lysates (right). **b** Growth curves. Left panel, MEFs. 3x10^5^ cells were placed in 6 cm dishes and numbers of cells were counted at the time points indicated. Right panel, SW480 derivatives. 2.5x10^4^ cells were placed into 6-wells and numbers of cells were counted at time points indicated. (n = 2 independent experiments, standard deviations are indicated). **c** Mean diameter and volume of *Htra1* ^*+/+*^ and *Htra1* ^*−/−*^ MEFs (unpaired two-tailed t-test, *p*-value <0.0001)

To investigate whether downregulation of HTRA1 might affect cell growth, we analysed the phenotypes of *Htra1*^+/+^ and *Htra1*^−/−^ MEFs generated from E14.5 mice that expressed either wild type levels of HtrA1 or no HtrA1. In parallel, we also generated a stable knockdown and overproduction of HTRA1 in SW480 cells, derived from a pool of clones, by using two independent shRNA constructs and plasmid p50, respectively. The knockout and knockdown of HTRA1 on mRNA level in MEFs and SW480 cells, respectively, was confirmed by qRT-PCR. While *Htra1*^*−/−*^ MEFs showed a 99.6 % depletion of *Htra1* mRNA the SW480 sh*HTRA1* cells had approximately 5 fold less *HTRA1* mRNA compared to the empty vector cells (Fig. 4a). Consequently, HTRA1 protein levels were decreased in *Htra1*^−/−^ MEFs and SW480 cells (Fig. 4a). Note that protein levels of SW480 cells were lower compared to MEFs. Therefore, cell culture supernatants were used for Western blotting to visualize protein levels of SW480 cells because levels of secreted HTRA1 are higher compared to cytoplasmic HTRA1 [1].

To explore the physiologic effects of HTRA1 depletion, we analysed growth curves to measure bulk proliferation rates in MEFs indicating that *Htra1*^−/−^ MEFs grew significantly faster compared to *Htra1*^*+/+*^ control cells, with a doubling time of 21 h compared to 38 h, respectively (Fig. 4b). In contrast, SW480sh*HTRA1* cells showed no significant differences in proliferation rates compared to the empty vector control (Fig. 4b), which is probably due to the transformed status of SW480 cancer cells. Since increased proliferation rates are often correlated with a reduction in cell size and volume [22], we determined cell volume and cell diameter in *Htra1*^*+/+*^ and *Htra1*^*−/−*^ MEFs. Indeed, the mean cell volume of *Htra1*^*−/−*^ MEFs was 2078 fl compared to 3323 fl of *Htra1*^*+/+*^ MEFs and the mean diameter of *Htra1*^*−/−*^ MEFs was 17 μm compared to 19 μm of *Htra1*^*+/+*^ MEFs (Fig. 4c). To determine any influence upon senescence, we serially passaged both *Htra1*^*+/+*^ and *Htra1*^*−/−*^ MEFs by splitting cells 1:3 twice per week. In 5 independent experiments, *Htra1*^*+/+*^ MEFs reached a senescence plateau after 12 passages (+/− 2), however *Htra1*^*−/−*^ MEFs continued to grow until passage 20 (+/− 2).

### Reduced HTRA1 expression drives polyploidy and correlates with centrosome amplification

**Fig. 5:**
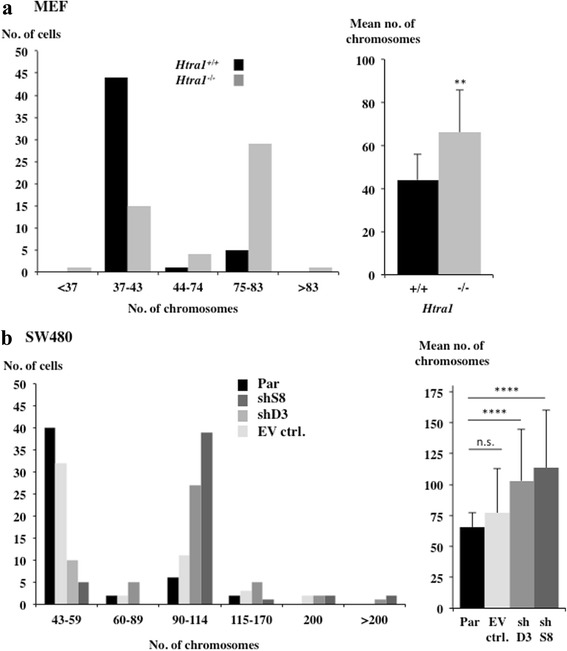
Increased polyploidy in *Htra1* ^*−/−*^ MEFs and SW480sh*HTRA1* cells. **a** Chromosome numbers of *Htra1* ^*−/−*^ and *Htra1* ^*+/+*^ MEFs. 50 mitotic cells were analysed by standard karyotyping. The normal set of chromosomes is 40. Shown is the distribution of chromosome numbers (left panel) as well as the mean number (right panel, two-tailed Mann–Whitney U test, *p*-value <0.01). **b** 50 mitotic SW480 cells were analysed by standard karyotyping. The normal set of chromosomes is 56–58. (Par, untransfected SW480; shS8 or D3, SW480 with *HTRA1* downregulated via two independent shRNAs; EV ctrl., corresponding to control carrying nonsense shRNA). The distribution of chromosome numbers (left panel) and the mean number (right panel, two-tailed Mann–Whitney U test, *p*-values: EV ctrl. = 0,7790, shD3 < 0.0001, shS8 < 0.0001) are shown

To address the question whether HTRA1 deficiency causes elevated genomic instability, karyotyping was performed to analyse chromosome numbers in *Htra1*^*+/+*^ and *Htra1*^*−/−*^ MEFs as well as in SW480 and derivatives of SW480 that were depleted for HTRA1 by using two independent stable shRNA constructs (Fig. 5). These data indicated that both cell lines exhibited polyploidy with the main peaks observed being 4n and 8n instead of 2n and a small peak of 4n in control cells (Fig. 5a, b). Note that SW480 cancer cells are already aneuploid [23].

**Fig. 6:**
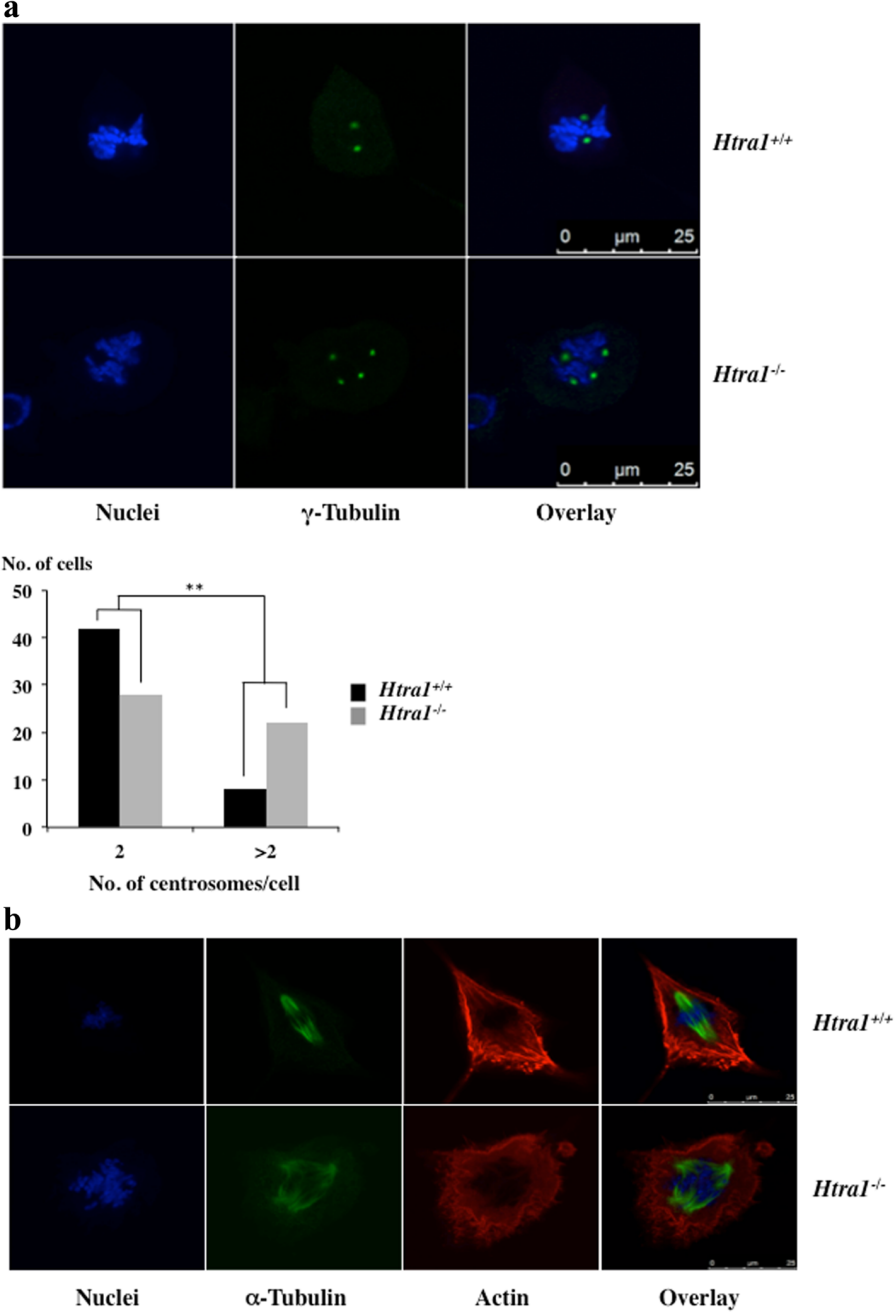
Disturbed spindle formation and centrosome abnormalities in *HTRA1* depleted cells. **a** Representative images of *Htra1* ^*+/+*^ and *Htra1* ^*−/−*^ MEFs. MEFs were grown on collagen coated glass slides, synchronised and analysed during mitosis by laser confocal microscopy. Centrosomes were visualised by anti-γ-tubulin antibody using an Alexa488 conjugated secondary antibody. Nuclei were counterstained with DAPI. Scale bar is 25 μm (upper panel). The number of centrosomes were counted in 50 *Htra1* ^*+/+*^ and *Htra1* ^*−/−*^ MEFs, respectively (lower panel). For statistical analysis, data were classified into two sets (2 and >2 centrosomes per cell) and analysed via Fisher’s exact test (*p*-value = 0.0041). **b** Representative images of immunofluorescence staining with an α-tubulin antibody using an Alexa488 conjugated secondary antibody in primary *Htra1* ^*+/+*^ and *Htra1* ^*−/−*^ MEFs (Passage 8). Nuclei were counterstained by DAPI. Scale bar is 25 μm

It has been shown previously that HTRA1 localises to centrosomes and microtubules in PC12 and SKOV3 cells [4, 12]. The cellular location of HTRA1 was confirmed for SW480 cells by analysing HTRA1-mCherry constructs using confocal microscopy (Additional file 1: Figure S1). Centrosomes normally nucleate microtubules to form the spindle that is subsequently used to position the chromosomes during cell division [24]. Since altered microtubule formation can predispose to polyploidy [25], we examined the location and number of centrosomes in mitotic cells by immunofluorescence staining using antibodies against gamma-tubulin in *Htra1*^*−/−*^ and *Htra1*^*+/+*^ MEFs. In the absence of HTRA1, an increase in the number of centrosomes (Fig. 6a) and multipolar spindles (Fig. 6b) were detected.

## Discussion

Several proteases such as members of the ADAMTS family, caspase 8 and trypsinogen IV are epigenetically silenced in cancer cells [26, 27]. As the *HTRA1* promoter contains a CpG island, we hypothesised that epigenetic mechanisms might be one way of lowering HTRA1 levels in cells. Our data indicate that, indeed, *HTRA1* is epigenetically repressed in the human colorectal cell line HCT116, but not in SW480 cells. In HCT116 cells, silencing is mediated by the methyl binding domain protein MBD2. In addition, the *Htra1* promoter is methylated and repressed in a proportion of benign polyps that develop in the *Apc*^*Min+*^ mouse model of human familial adenomatous polyposis. These data suggest that *Htra1* can be a target for epigenetic repression in intestinal neoplasia and that such targeting might be associated with the development of a subset of tumours. In this regard, it is significant that repression of *Htra1* is mediated by MBD2, the deficiency of which we have previously shown to strongly suppress the majority of adenomas in the *Apc*^*Min+*^ mouse [18]. The fact that adenomas do eventually form in this model suggests that MBD2-mediated suppression of target genes such as *Htra1* may be relevant to a subset of lesions. An implication of these studies is that *Htra1* status will influence adenoma formation in *Apc*^*Min+*^ mice, a hypothesis we are currently testing. Our findings may also have implications in the clinic, as the methylation state of the *HtrA1* promoter may act as a convenient biomarker for tumour cells or cells at risk of transformation.

To investigate the mechanistic consequences of *HTRA1* repression in tumorigenesis, we have analysed the phenotype of loss of function in both MEFs and SW480 cells. Remarkably, we find multiple phenotypes including increased proliferation, delayed onset of senescence, perturbed centrosome number and positioning, and ultimately polyploidy. Tight control of all aspects of cell division including accurate chromosome replication and partitioning as well as of proliferation rates is clearly critical to both tissue homeostasis and tumour suppression. Mechanistically, the changes we observe implicate a number of different pathways. The delayed onset of senescence suggests a failure in DNA damage checkpoint activation, as senescence can be triggered when telomeres are eroded and generate a DNA damage signal [28].

A second major phenomenon we observe is the deregulation of centrosomes and polyploidy. The perturbation of both centrosome position and number strongly implies a defect in microtubule function in the absence of HTRA1, as centrosomes normally nucleate microtubules to form the spindle, which is subsequently used to position the chromosomes during division. Such altered microtubules can predispose to polyploidisation. Thus, normal cells complete mitosis and enter S phase following activation of cyclin-dependent kinases in G_1_. However, if microtubule dynamics become perturbed, cells can aberrantly exit mitosis and enter S phase with a >4n DNA content, a process known as endoreduplication, ultimately resulting in polyploidy. This model is supported by the literature showing that HTRA1 is localised to microtubules and is a regulator of microtubule stability [4, 29, 30].

While these models offer initial explanations for some of the detected phenotypes of cells depleted for HTRA1, the underlying molecular mechanisms leading to increased proliferation rates, reduced cell size and the delayed senescence of primary *Htra1*^*−/−*^ MEFs remain to be identified. While it is likely that the loss of cell cycle checkpoint functions contributes, it is probably not the only reason. Due to the complexity of the cell cycle mechanism and of cellular transformation it will be interesting to address for example the interconnectivity of different checkpoints, the differential expression of HTRA1 in specific cell cycle phases and its role in regulating centrosome numbers and assembly in future experiments. Another key open question is the regulation of HTRA1 activity in mammalian cells. Previous studies revealed that HtrA proteases reversibly switch from the inactive into the active conformation and that this switch is mediated by specific peptidic ligands [1]. The identification of native modulators of HTRA1s activity (i.e. activators and inhibitors) and the exact circumstances under which these regulators are occurring will provide important insights into how this protease is implicated in the regulation of proliferation.

## Conclusions

These data show that MBD2-dependent epigenetic silencing of *HTRA1* can occur during tumour development. The phenotypes of reduced *HTRA1* expression such as acceleration of cell growth, centrosome amplification and polyploidy provide additional support for the model that proteolytic events are implicated in cancer biology. Moreover, the methylation state of the *HTRA1* promoter may be explored as a potential biomarker for tumour cells or cells at risk of transformation.

## Abbreviations

5-Aza-Dc, 5-Aza-2′Deoxycytidine; DNMT, DNA methyltransferase; HDAC, histone deacetylase; HtrA, High-temperature requirement A; MBD1, Methyl-CpG-binding domain protein 1; MBD2, Methyl Binding Domain protein 2; Me-CP2, Methyl-CpG binding protein 2; MEF, mouse embryonic fibroblasts; MNE, Mean normalized expression; TSA, Trichostatin A

## Additional file

Additional file 1: contains Supplementary method (Chromatin immunoprecipitation) and Table S1 (oligonucleotides used). Additional figure shows colocalisation of HTRA1 and microtubules in SW480 cells. (ZIP 394 kb)
