# Supplementary material for: Epigenetic silencing of serine protease HTRA1 drives polyploidy
Source: BMC Cancer. 2016 Jul 7;16:399. doi: 10.1186/s12885-016-2425-8 (PMC4936022; doi:10.1186/s12885-016-2425-8)
Supplement: Additional file 1: — contains Supplementary method (Chromatin immunoprecipitation) and Table S1 (oligonucleotides used). Additional figure shows colocalisation of HTRA1 and microtubules in SW480 cells. (ZIP 394 kb) [file 12885_2016_2425_MOESM1_ESM.zip › Additional data revisedR3.docx]

**Epigenetic Silencing of Serine Protease HTRA1 Drives Polyploidy**

Nina Schmidt^1,5^, Inga Irle^1,5^, Kamilla Ripkens^1^, Jasmin Nelles^1^, Christian Johannes^1^, Lee Parry^2^, Kirsty Greenow^2^, Sarah Amir^2^, Mara Campioni^3^, Alfonso Baldi^3^, Chio Oka^4^, Masashi Kawaichi^4^, Alan R Clarke^2^ and Michael Ehrmann^1,2*^

^1^ Centre for Medical Biotechnology, Faculty of Biology and Geography, University Duisburg-Essen, Universitaetsstrasse, D-45117 Essen, Germany

^2^ School of Biosciences, Cardiff University, Cardiff CF10 3US, UK

^3^ Department of Biochemistry and Biophysics, Section of Pathology, Second University of Naples, 80100 Naples, Italy

^4^ Division of Gene Function in Animals, Nara Institute of Science and Technology, 8916-5 Takayama, Ikoma, Nara 630-0192, Japan

^5^ These authors contributed equally to this work

**Running title** Epigenetic silencing of *HTRA1*

*^*^ Corresponding author: Michael Ehrmann, email:* [michael.ehrmann@uni-due.de](mailto:clausen@imp.univie.ac.at)

**Supplementary method**

**Chromatin immunoprecipitation (ChIP)**

Confluent SW480 and HCT116 cells were resuspended in pre-warmed PBS and crosslinked with PBS/1% formaldehyde for 15 min at RT. The reaction was stopped by adding glycine to a final concentration of 0.125 M for 5 min. All buffers were freshly supplemented with 1x protease inhibitor (Roche). Fixed cells were washed twice with ice-cold PBS, resuspended in lysis buffer (20 mM Tris pH 8.0, 85 mM KCL, 0.5% NP-40, 5 mM TSA), incubated on ice for 10 min and centrifuged (1300 g, 10 min, 4°C). Cell pellets were resuspended in nuclei lysis buffer (50 mM Tris pH 8.0, 10 mM EDTA, 1% SDS, 5 mM TSA) and incubated on ice for 10 min. Chromatin was sheared on ice by sonication using a Bandelin sonoplus HD2200 sonicator (28 cycles divided in 15s ON/60s OFF at 40% power with MS73 sonicator tip), followed by centrifugation (13.000 rpm, 10 min, 4°C) for collecting supernatants.

25 µg of sonicated chromatin was diluted 10 times in dilution buffer (20 mM Tris pH 8.0, 150 mM NaCl, 2 mM EDTA, 1% Triton X-100, 5 mM TSA) and pre-incubated for 1 h at 4°C with 20 µl of Protein A/G PLUS-Agarose (Santa Cruz) before the overnight incubation at 4°C with specific antibodies. For immunoprecipitation, 2 µg of RNApolII (Active Motif, No. 39097), IgG (Active Motif), H3 (Abcam, No. 1791), H3K9ac (Diagenode, pAB-177-050) and 10 µg MBD2a/b (Sigma, M7318) antibodies were used. Precipitates were collected by incubating 20 µl Protein A/G magnetic beads (Millipore, No. 16-663) for 4 h at 4°C and washed twice for 10 min at 4°C using 1 ml of buffers (low salt buffer: 20 mM Tris pH 8.0, 150 mM NaCl, 2 mM EDTA, 0.1% SDS, 1% Triton X-100; high salt buffer: 20 mM Tris pH 8.0, 500 mM NaCl, 2 mM EDTA, 0.1% SDS, 1% Triton X-100; LiCl buffer: 20 mM Tris pH 8.0, 250 mM LiCl, 1 mM EDTA, 1% NP-40, 1% sodiumdeoxycholate; TE buffer). Immune complexes were eluted twice in 150 µl elution buffer (1% SDS, 0.1 M NaHCO_3_) at 65°C for 15 min. Eluted samples were incubated overnight at 65°C, following incubation with ProteinaseK (Sigma) for 1 h and incubation with RNAseA (Sigma) for 30 min at 55°C. DNA was purified by using the Qiagen MinElute Purification Kit. qRT-PCR was used to determine the enrichment of immunoprecipitated DNA relative to the input material using gene-specific (HTRA1) and control (GAPDH) primer sets (Table S1).

**Table S1. Oligonucleotides**

| *DNA methylation* | *hHTRA1* | for GATTTGTAGGAATTTTTTTYGG |
| --- | --- | --- |
|  |  | rev ACCCAACCCATTAACCTC |
|  | *mHtra1* | for GAATTTTAGAATGAGAAATTGAGTTAGT |
|  |  | rev CAACAACAATAACAAAAACAAAAAC |
| sh*RNA* | sh*HTRA1*D3 | for CCGGgatctcaggagcgtatatattCTCGAGaatatatacgctcctggatcTTTTTG |
|  |  | rev AATTCAAAAAgatctcaggagcgtatatattCTCGAGaatatatacgctcctgagatc |
|  | sh*HTRA1*S8 | for CCGGcccaacagtttgcgccataaaCTCGAGtttatggcgcaaactgttgggTTTTTG |
|  |  | rev AATTCAAAAAcccaacagtttgcgccataaaCTCGAGtttatggcgcaaCtgttggg |
|  | sh*MBD2*.1 | for  CCGGGGAAGTGATCCGAAAATCTTTCTCGAGAAA |
|  | sh*MBD2*.1 | rev  AATTAAAAAGGAAGTGATCCGAAAATCTTTCTCGAGAAAGA |
|  | sh*MBD2*.2 | for  CCGGGAGCGATGTCTACTACTTCCTCGAGGAAGTAGTAGACATCGCTCTTTTTG |
|  | sh*MBD2*.2 | rev  AATTCAAAAAGAGCGATGTCTACTACTTCCTCGAGGAAGTAGTAGACATCGCTC |
|  | Nonsense RNA | for  CCGGCAACAAGATGAAGAGCACCAACTCGAGTTGGTGCTCTTCATCTTGTTGTTTTTG |
|  | Nonsense RNA | rev  AATTCAAAAACAACAAGATGAAGAGCACCAACTCGAGTTGGTGCTCTTCATCTTGTTG |
| *RT-qPCR* | mβ-Actin | for GATTACTGCTCTGGCTCCTAG |
|  |  | rev ACTCATCGTACTCCTGCTTGC |
|  | m*Gapdh* | for TTGATGGCAACAATCTCCAC |
|  |  | rev CGTCCCGTAGACAAAATGGT |
|  | *hGAPDH* | for GCTTGTCATCAATGGAAATCCC |
|  |  | rev AGCCTTCTCCATGGTGG |
|  | *hHTRA1* | for GCAACTCAGACATGGACTACATC |
|  |  | rev GTGTTAATTCCAATCACTTCACCG |
|  | *mHtra1* | for GTGCTCTCTGGGGCATATATC |
|  |  | rev ATGACATCGCTGACGTCATTG |
| *ChIP qPCR* | *hHTRA1*-453 | for GAACTTTCCCCGGCGC |
|  |  | rev GCGCCCCACATATTCGC |
|  | GAPDH-2 | Active motif, Cat. No. 71006 |
| *EMSA* | *HTRA1* | for CTCAG5-CH_3_-dCGAGAGA |
|  |  | rev TCTCT5-CH_3_-dCGCTGAG |
|  |  | for CTCAGCGAGAGA |
|  |  | rev TCTCTCGCTGAG |
|  | Control  [[1](#_ENREF_1)] | for GTATC5-CH_3_-dCGGATAC |
|  |  | rev GTATC5-CH_3_-dCGGATAC |
|  |  | for GTATCCGGATAC |
|  |  | rev GTATC CGGATAC |

**Supplementary reference**

1. Ohki I, Shimotake N, Fujita N, Jee J, Ikegami T, Nakao M, Shirakawa M: Solution structure of the methyl-CpG binding domain of human MBD1 in complex with methylated DNA. Cell 2001, 105(4):487-497.

**Supplementary figure legend**

**Figure S1. Microtubule association of HTRA1 in SW480 cells.** Cells were transiently transfected with HTRA1-mCherry (proteolytically inactive variant without signal sequence). Cells were analyzed via laser confocal microscopy. Nuclei were stained with DAPI and microtubules were visualized by anti-α-tubulin antibody (Invitrogen) using an Alexa488 conjugated secondary antibody (Molecular Probes).
