# Supplementary figures and images for: Epigenetic silencing of serine protease HTRA1 drives polyploidy
Source: BMC Cancer. 2016 Jul 7;16:399. doi: 10.1186/s12885-016-2425-8 (PMC4936022; doi:10.1186/s12885-016-2425-8)

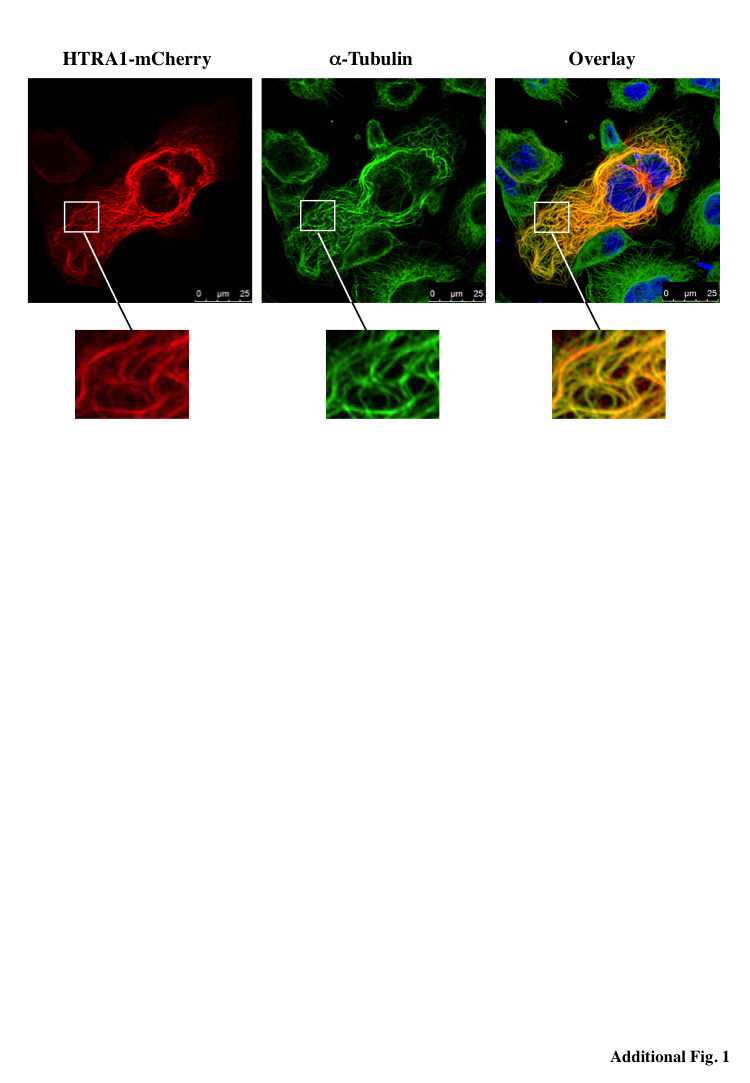

Supplement: Additional file 1: — contains Supplementary method (Chromatin immunoprecipitation) and Table S1 (oligonucleotides used). Additional figure shows colocalisation of HTRA1 and microtubules in SW480 cells. (ZIP 394 kb) [file 12885_2016_2425_MOESM1_ESM.zip › Additional Fig. S1R3.tif]
